# Spin-texture inversion in the giant Rashba semiconductor BiTeI

**DOI:** 10.1038/ncomms11621

**Published:** 2016-05-18

**Authors:** Henriette Maaß, Hendrik Bentmann, Christoph Seibel, Christian Tusche, Sergey V. Eremeev, Thiago R. F. Peixoto, Oleg E. Tereshchenko, Konstantin A. Kokh, Evgueni V. Chulkov, Jürgen Kirschner, Friedrich Reinert

**Affiliations:** 1 Experimentelle Physik VII, Universität Würzburg, Am Hubland, 97074 Würzburg, Germany; 2 Max-Planck-Institut für Mikrostrukturphysik, Weinberg 2, 06120 Halle, Germany; 3 Institute of Strength Physics and Materials Science, 634055 Tomsk, Russia; 4 Tomsk State University, 634050 Tomsk, Russia; 5 Saint Petersburg State University, 198504 Saint Petersburg, Russia; 6 Institute of Semiconductor Physics, 636090 Novosibirsk, Russia; 7 Novosibirsk State University, 636090 Novosibirsk, Russia; 8 Institute of Geology and Mineralogy, 630090 Novosibirsk, Russia; 9 Donostia International Physics Center (DIPC), 20018 San Sebastián/Donostia, Basque Country, Spain; 10 Departamento de Física de Materiales and Centro Mixto CSIC-UPV/EHU, Facultad de Ciencias Qumicas, Universidad del Pais Vasco/Euskal Herriko Unibertsitatea, Apdo. 1072, 20080 San Sebastián/Donostia, Basque Country, Spain

## Abstract

Semiconductors with strong spin–orbit interaction as the underlying mechanism for the generation of spin-polarized electrons are showing potential for applications in spintronic devices. Unveiling the full spin texture in momentum space for such materials and its relation to the microscopic structure of the electronic wave functions is experimentally challenging and yet essential for exploiting spin–orbit effects for spin manipulation. Here we employ a state-of-the-art photoelectron momentum microscope with a multichannel spin filter to directly image the spin texture of the layered polar semiconductor BiTeI within the full two-dimensional momentum plane. Our experimental results, supported by relativistic *ab initio* calculations, demonstrate that the valence and conduction band electrons in BiTeI have spin textures of opposite chirality and of pronounced orbital dependence beyond the standard Rashba model, the latter giving rise to strong optical selection-rule effects on the photoelectron spin polarization. These observations open avenues for spin-texture manipulation by atomic-layer and charge carrier control in polar semiconductors.

## Abstract (web summary)

In semiconductors containing heavy elements, the Rashba spin-orbit interaction can couple the momentum and spin of electrons, yielding spintronic functionality. Here, the authors image band- and orbital-dependent spin-textures in the layered polar semiconductor BiTeI, demonstrating behaviour beyond the standard Rashba model.

## Graphical abstract

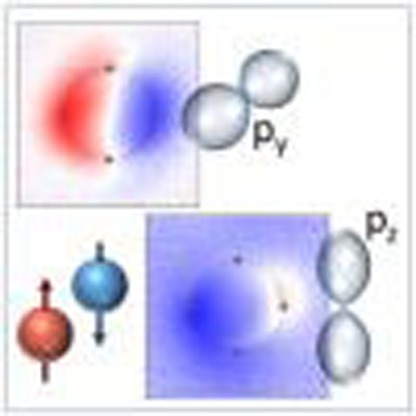

The coupling between spin and orbital degrees of freedom has emerged as a crucial feature in the electronic structure of semiconductors and low-dimensional materials^1^,^2^,^3^. The fundamental influence of spin–orbit interaction on the electronic wave functions gives rise to topologically protected surface states and to characteristic spin textures, in both real and momentum space. Making use of these effects to control the electron spin is a central goal in spintronics. In particular, the Rashba effect^4^, allowing for spin manipulation via electric fields, is often used in proposed devices, such as spin-field and spin Hall effect transistors^5^,^6^,^7^,^8^. Rashba-type spin–orbit interaction induces chiral spin textures in the electronic structure by coupling the electron momentum and spin degrees of freedom^9^. Identifying materials with large spin splittings where the spin texture at the Fermi surface can be modified by external parameters will introduce enhanced versatility in controlling spin-dependent transport properties. Recent proposals in this direction include, for example, ferroelectric semiconductors where spin–orbit coupling is influenced by the switchable electric polarization^10^,^11^.

Layered van-der-Waals materials are studied extensively due to their extraordinary bulk and surface electronic properties, which are considered to be promising for various technological applications^12^,^13^. Strong spin–orbit effects can be achieved in these compounds by incorporation of heavy atoms, such as in Bi_2_Te_3_ and Bi_2_Se_3_ that realize topological insulators with non-trivial spin-polarized surface states^3^. A related class of layered semiconductors is represented by BiTeI whose polar bulk crystal structure is built of ionically bound (BiTe)^+^ and I^−^ layers. The resulting electric polarization along the layer stacking axis facilitates a giant Rashba effect in the electronic structure of BiTeI and related materials^14^,^15^,^16^,^17^. It also gives rise to a pronounced n-type or p-type band bending at the surface in dependence of the nominal charge of the terminating atomic layer^18^,^19^. This makes it possible to place either valence or conduction band states at the Fermi level by choice of the surface termination^18^.

In this work we directly image the full momentum-dependent spin textures of the valence and conduction band-derived states in BiTeI by the use of spin-resolved photoelectron momentum microscopy, a highly efficient technique for determining spin-dependent band structures in solids that only recently has become available^20^,^21^,^22^. Surprisingly, our measurements reveal opposite spin chiralities for electronic states at the valence band top and at the conduction band bottom, despite the fixed polar electric field vector determined by the (BiTe)^+^-I^−^ stacking sequence. This implies the possibility to modify the spin texture through control of the charge carrier type, which, in BiTeI, could be realized through n-type and p-type band bending in dependence of surface termination^18^. Our observations strongly support a previous theory that predicts Rashba parameters of opposite sign in the conduction and valence band as a generic feature in semiconductors with giant spin–orbit interaction^23^. Furthermore, our measurements provide evidence for an additional orbital dependence of the Rashba effect in these materials^24^,^25^. This manifests itself in pronounced changes of the measured photoelectron spin textures depending on the polarization of the exciting light, which are in sharp contrast to the metallic Rashba system Au(111)^22^,^26^ but in remarkable correspondence to recent findings in the topological insulator Bi_2_Se_3_ (refs 26, 27).

## Results

### Rashba splitting of conduction and valence band states in BiTeI

**Figure 1:**
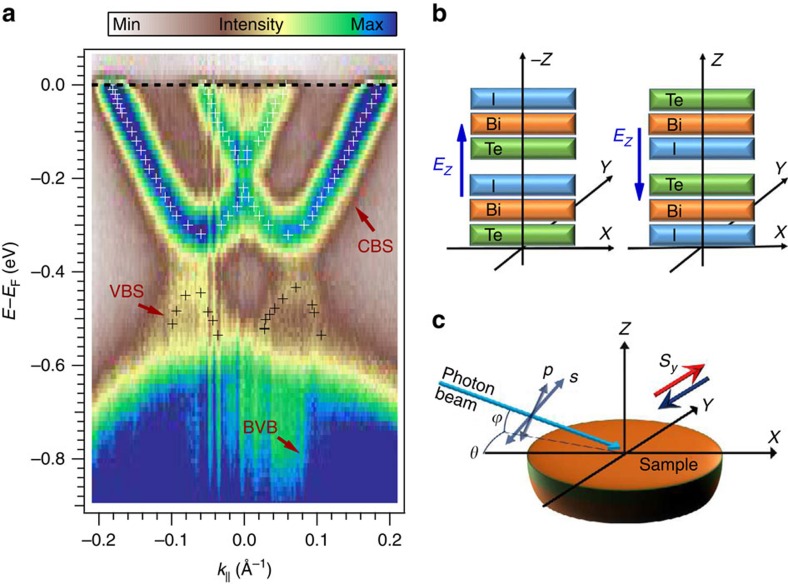
Giant Rashba splitting in BiTeI. (**a**) Angle-resolved photoemission spectroscopy (ARPES) data of BiTeI showing two surface states that develop from the valence band (VBS) and conduction band (CBS), and exhibit large Rashba-type spin splittings. Black and white markers indicate estimated peak maxima for both bands. Additional spectral weight due to bulk valence bands (BVB) is visible at lower energies. (**b**) Schematic of the two possible atomic stacking orders in non-centrosymmetric BiTeI. The inversion of the polar layer stacking corresponds to a reversal of the crystalline *z* axis and of the built-in electric field *E*_*z*_, generating the Rashba splitting. The natural cleaving planes of the crystal lie between Te and I layers, giving rise to I- and Te-terminated surfaces. (**c**) Sketch of the experimental geometry for spin-resolved measurements of the spin component *S*_*y*_ (blue and red arrow). For *p*-polarized light the light electric field is oriented parallel to the plane given by surface normal and incoming light, whereas for *s*-polarized light it is oriented perpendicular to the plane of incidence.

Our analysis concentrates on the two surface states shown in Fig. 1a. Both states host a large Rashba-type spin splitting demonstrated by the black and white markers that trace the dispersion of the states. The upper band derives from the conduction band and has a positive effective mass 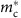; the second band, with a negative effective mass 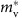, develops from the valence band. The conduction and valence band surface states have been shown to be located on Te- and I-terminated surface areas, respectively. The latter correspond to two types of stacking-fault-induced domains in the bulk of BiTeI in which the atomic stacking order and thus the crystalline *z* axis are inverted, as illustrated in Fig. 1b (refs 18, 28). Owing to the presence of both domain types in the bulk, the natural cleavage plane between Te and I layers exposes Te- and I-terminated areas on the same surface. Both terminations occur to the same extent on the sample surface with domain widths of 10–100 nm (refs 19, 28, 29). The momentum microscope employed for our experiments probes a sample area of ∼20 μm in diameter. Therefore, in our measurements we are able to probe both states simultaneously.

### Spin-texture inversion and sign change of the Rashba parameter

**Figure 2:**
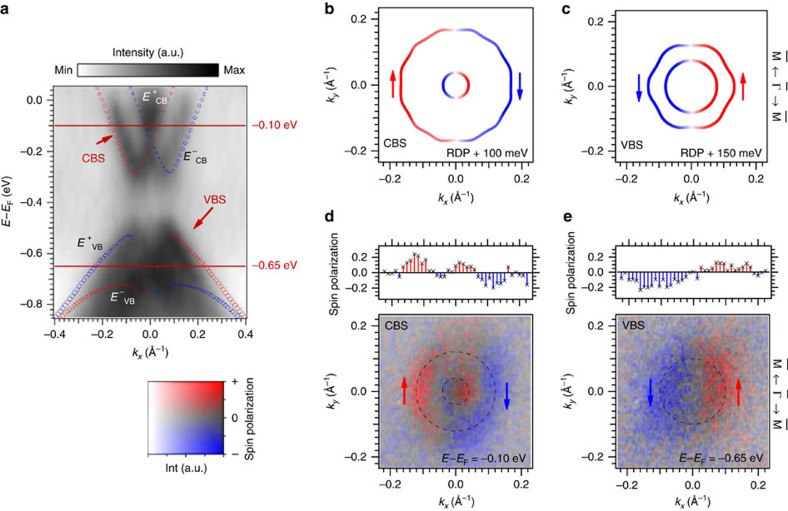
Spin-texture inversion between valence and conduction band surface states. (**a**) Band structure of BiTeI measured with *hν*=21.2 eV. Small open circles show the calculated dispersions 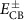 and 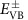 of the conduction band and valence band Rashba-split surface states (CBS/VBS), resided at the Te and I terminations, respectively. The calculated binding energies have been adjusted to match the experimental data. Solid red lines indicate the energies at which the spin-resolved measurements (**d**,**e**) were performed. The constant energy contours for the conduction band (**b**) and valence band (**c**) surface states are calculated at energies of 100 and 150 meV above the Rashba degeneracy points (RDPs), respectively. The measured spin component is aligned parallel to the *y* direction with blue marking negative and red marking positive spin polarization as indicated by the red and blue arrows in **b**–**e**, while colour strength denotes the photoemission intensity.

We performed band structure calculations based on density functional theory, to capture the ground-state spin polarization in the surface electronic structure. The calculated spin-resolved Rashba-split surface state dispersions for the Te- and I-terminated BiTeI surfaces are shown in Fig. 2a by open circles superimposed onto the experimentally obtained electronic structure. The calculated binding energies were adjusted individually for the two terminations, to match the respective experimental binding energies. Calculated constant energy contours for the conduction band surface state and valence band surface state are displayed in Fig. 2b,c, respectively. Here, the red and blue colours indicate the positive/negative sign of the *S*_*y*_ cartesian spin component. The conduction band surface state (Fig. 2b) and the valence band surface state (Fig. 2c) show a circular inner structure and a slightly warped outer one. Between the outer sub-bands of the two states, a reversal of the spin polarization occurs. More precisely, we find a clockwise spin chirality in the outer sub-band of the conduction band state and a anti-clockwise chirality in the outer branch of the valence band state. The inner contour of the conduction band state shows a reversed spin polarization compared with the outer one, whereas for the valence band state both branches have the same spin polarization as expected for energies above the Rashba degeneracy point.

The theoretical result is nicely reproduced by our measured spin polarization obtained with an imaging spin filter installed in a momentum microscope. The data are shown in Fig. 2d,e where the red (blue) colour code once again represents the spin polarization along the *y* axis. The conduction band state was probed at a binding energy of 100 meV as illustrated by the red horizontal line in Fig. 2a. The measured spin polarization is highest along the two dotted circles in Fig. 2d, which indicate the two sub-bands of the spin-split state. It is reversed between the outer and inner circle, reflecting an opposite spin chirality of the two branches in line with the general Rashba picture. The switch of the spin polarization between the two branches can be inferred in more detail from the polarization plot along the wave vectors *k*_*x*_ at *k*_*y*_=0 Å^−1^ shown above the momentum map. The spin polarization is opposite at ∼+0.12 and −0.12 Å^−1^, which corresponds to the outer branch. It also switches between ∼+0.04 and −0.04 Å^−1^ where the inner structure lies.

The spin polarization of the valence band state, measured at a binding energy of 650 meV, is shown in Fig. 2e. Black dotted circles serve as guides to the eye. The data show an opposite spin texture compared with the outer branch of the conduction band state. The spin polarization along *k*_*x*_ shown above the colour plot reveals the sign switch at ∼*k*_*x*_=0 Å^−1^ from negative to positive polarization.

Based on the excellent agreement between the experimental and calculated spin chiralities, we will now discuss the relative sign of the spin textures in the probed bands. In general, the Rashba splitting in BiTeI originates from the polar atomic-layer stacking along the crystalline *z* direction that gives rise to a net electric field *E*_*z*_ across the unit cell, as sketched in Fig. 1b. This holds for the Rashba splitting of the bulk electronic bands but also for one of the surface bands^17^. The latter split off in energy from the respective bulk bands due to modifications in the near-surface potential and thereby inherit the Rashba splitting and spin texture from the bulk states. For the present measurements it is therefore important to take into consideration that the two probed surface states are located in domains with reversed atomic stacking order, which inverts the electric field *E*_*z*_ generating the Rashba splitting (see Fig. 1b). This reversal of the crystalline *z* axis in one of the domains naturally implies an inversion of the spin textures of electronic states located in this domain^16^, which needs be taken into account in a comparison between the two states.

Within the Rashba model the spin splitting is described by the Rashba parameter *α*_R_. Its magnitude determines the size of the splitting, whereas its sign *α*_R_/|*α*_R_| defines the spin chirality^30^. More precisely, the sign of *α*_R_ determines whether the upper branch *E*^+^ of a Rashba split band has anti-clockwise or clockwise spin chirality and conversely for the lower branch *E*^−^. Therefore, by determining the chiralities in the valence and conduction band states, one can identify the relative sign between the Rashba parameters that characterize the conduction band bottom and valence band top. Doing so in Fig. 2 we find, in both the experiment and the calculation, that the upper branch of the conduction band state 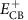, located in a Te-terminated domain, and the upper branch of the valence band state 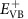, located in an I-terminated domain, have the same chirality. Taking into account the spin-texture reversal originating from the opposite orientation of the crystalline *z* axis in the two domains, we thus infer opposite Rashba parameters for the valence band state and the conduction band state.

In a simplified picture where the Rashba parameter is proportional to the symmetry-breaking electric field, *α*_R_∝*E*_*z*_, this result may appear surprising. However, model calculations predicted that Rashba parameters of opposite sign in the topmost valence and lowest conduction band are indeed a generic feature of the band structure of BiTeI^23^, in agreement with the present observations. In fact, it is the coupling between these two bands, being mediated by spin–orbit interaction, which gives rise to Rashba parameters of large magnitude and of opposite sign.

It is noteworthy that in the experimental data of the valence band state (Fig. 2e) the outer and inner branch cannot be resolved due to the fact that the measurement was taken at an energy above the Rashba degeneracy point where the spin polarization should have the same sign. In addition, the I termination is highly liable to ageing effects that gradually shift the state to higher binding energies during the timescale of the experiment^28^,^29^, which leads to a smearing out of the measured spin polarization. We therefore show a calculation at 150 meV above the Rashba degeneracy point for the valence band surface state in Fig. 2c, which represents the spin polarization in the measured energy range. The ageing effect also explains the differences between the measured surface states shown in Figs 1a and 2a.

### Orbital dependence of the spin texture

To gather insight into the interplay between the electron spin and the involved orbitals, we performed an additional set of spin-resolved measurements on the conduction band state where we used linearly polarized light with a photon energy of *hν*=6 eV. Switching the polarization from *s*- to *p*-polarization allows to address different *p*-orbitals, as the photoemission dipole matrix element 
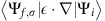
 changes with light polarization 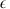. The initial state wave function along *k*_*x*_ can be written as 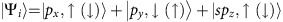, where the spin ↑, ↓ is quantized along the *y* axis^31^,^32^. It follows that *s*-polarized light mainly excites electrons from the *p*_*y*_ orbital, while the *p*_*x*_ and *p*_*z*_ orbitals are excited by *p*-polarized light^33^. A strong dependence of the photoelectron spin polarization on the polarization of the incoming light has been predicted for different spin–orbit coupled materials^34^,^35^ and shown for topological surface states^26^,^27^,^36^ and for the case of unpolarized ground states^37^,^38^.

**Figure 3:**
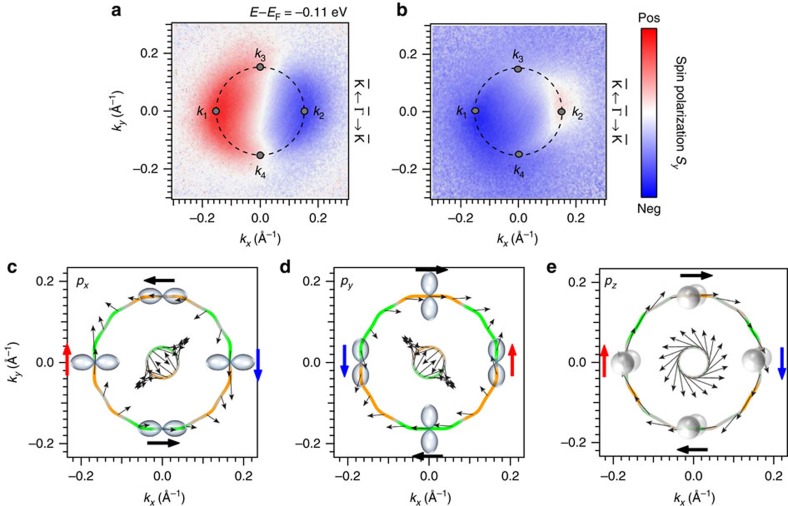
Orbital-dependent spin texture of BiTeI. Spin polarization maps at 110 meV binding energy acquired with (**a**) *s*-polarized light and (**b**) *p*-polarized light, using a photon energy of *hν*=6 eV. Red and blue colours signal a spin polarization pointing along the positive/negative *y* axis. The surface state is highlighted with dotted circles and grey dots indicate points *k*_1−4_ in momentum space for which the spin polarization is discussed below. Calculated projections of the total spin on the (**c**) *p*_*x*_, (**d**) *p*_*y*_ and (**e**) *p*_*z*_ orbital. Here, arrows indicate the in-plane components, while green (orange) colour represents the negative (positive) spin polarization along *z*.

Indeed, the results of the measurements that are shown in Fig. 3a,b clearly shows a different spin texture when the light polarization is changed. The two-dimensional (2D) photoelectron spin texture is almost symmetric when measured with *s*-polarized light, whereas a large asymmetry occurs for *p*-polarized light. This corresponds to the light polarization 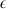, which obeys mirror symmetry along ±*k*_*x*_ for *s*-polarization but breaks mirror symmetry for *p*-polarization. When measured with *s*-polarized light, the detected spin polarization *S*_*y*_ switches from positive (red) at −*k*_*x*_ to negative (blue) at +*k*_*x*_. At ∼*k*_*x*_=0 Å^−1^, the spin polarization becomes zero. It is noteworthy that only the outer branch is resolved, which we attribute to bulk bands that overlap with the surface state at small *k*_||_ (ref. 15). For the measurement with *p*-polarized light, the situation is different; in particular the spin polarization at −*k*_*x*_ is negative, whereas we find a positive spin polarization at positive *k*_*x*_ values. This result already provides evidence for a coupling between the spin and the different *p*-orbitals.

To understand precisely how this coupling is realized, we analyse the calculated cartesian spin components projected onto the *p*_*x*_, *p*_*y*_ and *p*_*z*_ orbitals as shown in Fig. 3c,d, respectively. Here, the black arrows adjacent to the contours indicate the in-plane spin polarization, while orange and green colours stand for the positive/negative sign of the *S*_*z*_ component. The in-plane spin polarization at points along high-symmetry directions is additionally highlighted by bold arrows. The corresponding points in momentum space are marked by grey dots in the experimental data and labelled with *k*_1_–*k*_4_.

Along 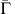
–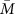 at *k*_1_ and *k*_2_, the calculated in-plane spin polarization is aligned parallel to the *y* axis. The spin polarization in the *p*_*y*_ orbital has opposite sign when compared with the *p*_*x*_ and the *p*_*z*_ orbital at these points. This corresponds to the experimental data where *S*_*y*_ has opposite sign for *s*- and *p*-polarized light. At *k*_3_ and *k*_4_, which lie along 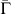
−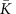, the calculated ground-state spin polarization has an in-plane component *S*_*x*_ parallel to the *x* axis, which has the same sign for the *p*_*y*_ and *p*_*z*_ orbital and is reversed for the *p*_*x*_ orbital.

A similar photoelectron spin-texture inversion between *s*- and *p*-polarized light has been observed for the spin-polarized topological surface state on Bi_2_Se_3_ (refs 26, 27, 39, 40). It is absent, however, for the Rashba-split surface state of Au(111)^22^,^26^, a longstanding paradigm for spin–orbit splitting at surfaces^41^,^42^. It has therefore been unclear whether such manipulation of the photoelectron spin polarization is a generic feature due to spin–orbit coupling or related also to material-specific details of Bi_2_Se_3_, as in particular its non-trivial topology^43^. The present experimental results show that topology plays no significant role, and that very strong spin–orbit interaction, as introduced by heavy elements as for instance Bi, may be necessary to generate sufficient spin–orbital mixing.

In the experimental data we find a spin polarization close to zero at *k*_3_ and *k*_4_ for the *s*-polarized light. Therefore, no *S*_*y*_ contribution is measured in line with the calculated result for *p*_*y*_. For the *p*-polarized light on the other hand, a spin polarization signal *S*_*y*_ of equal sign is measured at *k*_3_ and *k*_4_. This spin component parallel to the wave vector differs from the ground-state calculations and the general expectation from a simple Rashba picture. Radial components in the spin polarization have, for instance, been measured in the topological insulator Bi_2_Se_3_ and explained by a layer-dependent interference effect between different orbital contributions during the photoemission process^27^,^44^.

It is worth noting that the photoelectron spin polarization measured with an unpolarized light source with a photon energy of *hν*=21.2 eV (shown in Fig. 2d) has the same sign as the spin polarization measured at *hν*=6 eV with *s*-polarized light. For unpolarized light—at least for metallic surfaces—we expect a *p*-like polarization inside the sample according to the Fresnel equations^45^. This result therefore suggests a possible photon energy dependence of the spin polarization. The spin-up and spin-down parts of the wave function are coupled to different spatial parts with possibly different photon energy-dependent cross-sections. Therefore, such a change in the spin polarization, even for the same light polarization, is in general conceivable^46^,^47^. A dependence of the photoelectron spin polarization on photon energy has, for instance, been predicted for layered spin–orbital textures and certain geometries^27^,^48^, and was also shown in the case of circularly polarized light^36^. A complete switch of the spin polarization has on the other hand not been experimentally established.

## Discussion

Our polarization-dependent measurements establish the concept of orbital-selective spin textures in 2D momentum space in non-topological Rashba-split electron systems. This is important for possible opto-spintronic applications and from a conceptual point of view^26^,^43^. It is also of particular relevance in the context of other materials where spin and orbital textures and their momentum-dependent coupling are currently widely discussed, such as topological crystalline insulators^49^, transition metal dichalcogenides^50^,^51^ and transition metal oxides^52^, with importance, for example, for electron scattering and optical excitation phenomena. In particular, in the case of giant Rashba semiconductors the orbital dependence may open additional possibilities to modify the spin textures, for example, through photoexcitation as shown in the present work.

Furthermore, the observation of opposite spin chiralities for the conduction and valence band electrons in BiTeI opens pathways to manipulate the spin polarization through choice of the charge carrier type. In particular, in BiTeI and related polar semiconductors this can be realized via different surface terminations and thus through control of electrostatic fields at the surface or interface^18^. Interestingly, lateral p–n junctions, recently imaged in BiTeI by scanning tunnelling microscopy^29^, could therefore provide the opportunity to not only switch the carrier type but also the spin chirality at the Fermi level. In general, the band- and orbital-selective spin textures reported here for BiTeI provide degrees of freedom to control spin-polarized electronic states in polar semiconductors.

## Methods

### Experiment

The spin-resolved angle-resolved photoemission spectroscopy experiments were performed using a spin-resolving momentum microscope, which is able to simultaneously capture the complete 2D momentum space^22^. An additional imaging spin filter installed behind the *k*-resolving optics exploits the spin-selective mirror-like reflection of the photoelectrons at a gold passivated iridium surface^20^ to measure the *y*-component of the photoelectron spin polarization. A non-monochromatized He-discharge lamp (21.2 eV) and the fourth harmonic of a Ti:Sa (6 eV) oscillator served as light sources. The measurement geometry is shown in Fig. 1c, where Θ=30° and *φ*=22° for the He-light source and Θ=0° and *φ*=8° for the Ti:Sa oscillator. The energy resolution in the spin-resolved momentum maps was set to 80 meV for measurements with the He light source (Fig. 2) and 20 meV for the laser light source (Fig. 3). All experiments were performed at *T*=130 K and at pressures of the order of *p*=10^−10^ mbar. The spin-resolved data were processed as described in refs 22, 21. Single crystals of BiTeI were prepared by *in situ* cleaving with adhesive tape at pressures below 10^−9^ mbar. The samples have been grown by a modified Bridgman method with rotating heat field as described in refs 28, 53.

### Theory

For electronic band structure calculations, we employed DFT with the generalized gradient approximation^54^ for the exchange correlation potential as implemented in the Vienna *Ab Initio* Simulation Package^55^,^56^. The interaction between the ion cores and valence electrons was described by the projector augmented-wave method^57^,^58^. The Hamiltonian contained the scalar relativistic corrections and the spin–orbit coupling was taken into account by the second variation method. For the simulation of the BiTeI surfaces, we used 24 atomic layer slabs that were terminated on one side by a monolayer of hydrogen to clearly separate the surface states localized on opposite sides of the slab.

## Additional information

**How to cite this article:** Maaß, H. *et al.* Spin-texture inversion in the giant Rashba semiconductor BiTeI. *Nat. Commun.* 7:11621 doi: 10.1038/ncomms11621 (2016).

## References

[b1] Manchon A., Koo H. C., Nitta J., Frolov S. M. & Duine R. A. New perspectives for Rashba spin-orbit coupling. Nat. Mater. 14, 871–882 (2015). 26288976 10.1038/nmat4360

[b2] Xu X., Yao W., Xiao D. & Heinz T. F. Spin and pseudospins in layered transition metal dichalcogenides. Nat. Phys. 10, 343–350 (2014).

[b3] Hasan M. Z. & Kane C. L. *Colloquium* : topological insulators. Rev. Mod. Phys. 82, 3045–3067 (2010).

[b4] Bychkov Y. A. & Rashba E. I. Properties of a 2D electron gas with lifted spectral degeneracy. JETP Lett. 39, 66–69 (1984).

[b5] Datta S. & Das B. Electronic analog of the electro-optic modulator. Appl. Phys. Lett. 56, 665 (1990).

[b6] Koo H. C. *et al.* Control of spin precession in a spin-injected field effect transistor. Science 325, 1515–1518 (2009). 19762637 10.1126/science.1173667

[b7] Wunderlich J. *et al.* Spin Hall effect transistor. Science 330, 1801–1804 (2010). 21205664 10.1126/science.1195816

[b8] Schliemann J., Egues J. C. & Loss D. Nonballistic spin-field-effect transistor. Phys. Rev. Lett. 90, 146801 (2003). 12731937 10.1103/PhysRevLett.90.146801

[b9] Winkler R. in Springer Tracts in Modern Physics Vol. 191, (Springer (2003).

[b10] Kim M., Im J., Freeman A. J., Ihm J. & Jin H. Switchable S=1/2 and J=1/2 Rashba bands in ferroelectric halide perovskites. Proc. Natl Acad. Sci. 111, 6900–6904 (2014). 24785294 10.1073/pnas.1405780111 PMC4024856

[b11] Di Sante D., Barone P., Bertacco R. & Picozzi S. Electric control of the giant rashba effect in bulk GeTe. Adv. Mater. 25, 509–513 (2013). 23070981 10.1002/adma.201203199

[b12] Butler S. Z. *et al.* Progress, challenges, and opportunities in two-dimensional materials beyond graphene. ACS Nano 7, 2898–2926 (2013). 23464873 10.1021/nn400280c

[b13] Crepaldi A. *et al.* Momentum and photon energy dependence of the circular dichroic photoemission in the bulk Rashba semiconductors BiTe X (X=I , Br, Cl). Phys. Rev. B 89, 125408 (2014).

[b14] Ishizaka K. *et al.* Giant Rashba-type spin splitting in bulk BiTeI. Nat. Mater. 10, 521–526 (2011). 21685900 10.1038/nmat3051

[b15] Sakano M. *et al.* Strongly spin-orbit coupled two-dimensional electron gas emerging near the surface of polar semiconductors. Phys. Rev. Lett. 110, 107204 (2013). 23521291 10.1103/PhysRevLett.110.107204

[b16] Landolt G. *et al.* Disentanglement of surface and bulk rashba spin splittings in noncentrosymmetric BiTeI. Phys. Rev. Lett. 109, 116403 (2012). 23005655 10.1103/PhysRevLett.109.116403

[b17] Eremeev S. V., Nechaev I. A., Koroteev Y. M., Echenique P. M. & Chulkov E. V. Ideal two-dimensional electron systems with a giant Rashba-type spin splitting in real materials: surfaces of bismuth tellurohalides. Phys. Rev. Lett. 108, 246802 (2012). 23004307 10.1103/PhysRevLett.108.246802

[b18] Crepaldi A. *et al.* Giant ambipolar Rashba effect in the semiconductor BiTeI. Phys. Rev. Lett. 109, 096803 (2012). 23002871 10.1103/PhysRevLett.109.096803

[b19] Butler C. J. *et al.* Mapping polarization induced surface band bending on the Rashba semiconductor BiTeI. Nat. Commun. 5, 4066 (2014). 24898943 10.1038/ncomms5066 PMC4059917

[b20] Vasilyev D. *et al.* Low-energy electron reflection from Au-passivated Ir(001) for application in imaging spin-filters. J. Electron. Spectrosc. Relat. Phenom. 199, 10–18 (2015).

[b21] Tusche C. *et al.* Quantitative spin polarization analysis in photoelectron emission microscopy with an imaging spin filter. Ultramicroscopy 130, 70–76 (2013). 23561302 10.1016/j.ultramic.2013.02.022

[b22] Tusche C., Krasyuk A. & Kirschner J. Spin resolved bandstructure imaging with a high resolution momentum microscope. Ultramicroscopy 159, 520–529 (2015). 25840475 10.1016/j.ultramic.2015.03.020

[b23] Bahramy M. S., Arita R. & Nagaosa N. Origin of giant bulk Rashba splitting: application to BiTeI. Phys. Rev. B 84, 041202 (R) (2011).

[b24] Zhu Z., Cheng Y. & Schwingenschlögl U. Orbital-dependent Rashba coupling in bulk BiTeCl and BiTeI. New J. Phys. 15, 023010 (2013).

[b25] Bawden L. *et al.* Hierarchical spin-orbital polarization of a giant Rashba system. Sci. Adv. 1, e1500495 (2015). 26601268 10.1126/sciadv.1500495 PMC4643772

[b26] Jozwiak C. *et al.* Photoelectron spin-flipping and texture manipulation in a topological insulator. Nat. Phys. 9, 293–298 (2013).

[b27] Zhu Z.-H. *et al.* Photoelectron spin-polarization control in the topological insulator Bi_2_Se_3_. Phys. Rev. Lett. 112, 076802 (2014). 24579623 10.1103/PhysRevLett.112.076802

[b28] Fiedler S. *et al.* Defect and structural imperfection effects on the electronic properties of BiTeI surfaces. New J. Phys. 16, 075013 (2014).

[b29] Tournier-Colletta C. *et al.* Atomic and electronic structure of a Rashba p-n junction at the BiTeI surface. Phys. Rev. B 89, 085402 (2014).

[b30] Henk J., Hoesch M., Osterwalder J., Ernst A. & Bruno P. Spin-orbit coupling in the L-gap surface states of Au(111): spin-resolved photoemission experiments and first-principles calculations. J. Phys. Condens. Matter 16, 7581–7597 (2004).

[b31] Mirhosseini H. *et al.* Unconventional spin topology in surface alloys with Rashba-type spin splitting. Phys. Rev. B 79, 245428 (2009).

[b32] Bentmann H., Abdelouahed S., Mulazzi M., Henk J. & Reinert F. Direct observation of interband spin-orbit coupling in a two-dimensional electron system. Phys. Rev. Lett. 108, 196801 (2012). 23003070 10.1103/PhysRevLett.108.196801

[b33] Cao Y. *et al.* Mapping the orbital wavefunction of the surface states in three-dimensional topological insulators. Nat. Phys. 9, 499–504 (2013).

[b34] Henk J., Ernst A. & Bruno P. Spin polarization of the L-gap surface states on Au(111). Phys. Rev. B 68, 165416 (2003).

[b35] Wissing S. *et al.* Ambiguity of experimental spin information from states with mixed orbital symmetries. Phys. Rev. Lett. 113, 116402 (2014). 25259990 10.1103/PhysRevLett.113.116402

[b36] Sánchez-Barriga J. *et al.* Photoemission of Bi_2_Se_3_ with circularly polarized light: probe of spin polarization or means for spin manipulation? Phys. Rev. X 4, 011046 (2014).

[b37] Tamura E., Piepke W. & Feder R. New spin-polarization effect in photoemission from nonmagnetic surfaces. Phys. Rev. Lett. 59, 934–937 (1987). 10035910 10.1103/PhysRevLett.59.934

[b38] Schneider C. M., Garbe J., Bethke K. & Kirschner J. Symmetry-dependent alignment of the electron-spin polarization vector due to electronic band hybridization observed in photoemission from Ag (100). Phys. Rev. B 39, 1031 (1989). 10.1103/physrevb.39.1031 9948282

[b39] Zhang H., Liu C.-X. & Zhang S.-C. Spin-orbital texture in topological insulators. Phys. Rev. Lett. 111, 066801 (2013). 23971598 10.1103/PhysRevLett.111.066801

[b40] Xie Z. *et al.* Orbital-selective spin texture and its manipulation in a topological insulator. Nat. Commun. 5, 3382 (2014). 24584220 10.1038/ncomms4382

[b41] LaShell S., McDougall B. A. & Jensen E. Spin splitting of an Au (111) surface state band observed with angle resolved photoelectron spectroscopy. Phys. Rev. Lett. 77, 3419 (1996). 10062215 10.1103/PhysRevLett.77.3419

[b42] Nicolay G., Reinert F., Hüfner S. & Blaha P. Spin-orbit splitting of the *L* -gap surface state on Au(111) and Ag(111). Phys. Rev. B 65, 033407 (2001).

[b43] Xue Q.-K. Topological insulators: full spin ahead for photoelectrons. Nat. Phys. 9, 265–266 (2013).

[b44] Jozwiak C. *et al.* Widespread spin polarization effects in photoemission from topological insulators. Phys. Rev. B 84, 165113 (2011).

[b45] Maniv T. & Metiu H. Electrodynamics at a metal surface. II. Fresnel formulas for the electromagnetic field at the interface for a jellium model within the random phase approximation. J. Chem. Phys. 76, 2697 (1982).

[b46] Krasovskii E. E. Spin-orbit coupling at surfaces and 2d materials. J. Phys. Condens. Matter 27, 493001 (2015). 26580290 10.1088/0953-8984/27/49/493001

[b47] Heinzmann U. & Dil J. Spin-orbit-induced photoelectron spin polarization in angle-resolved photoemission from both atomic and condensed matter targets. J. Phys. Condens. Matter 24, 173001 (2012). 22480989 10.1088/0953-8984/24/17/173001

[b48] Zhu Z.-H. *et al.* Layer-by-layer entangled spin-orbital texture of the topological surface state in Bi_2_Se_3_. Phys. Rev. Lett. 110, 216401 (2013). 23745898 10.1103/PhysRevLett.110.216401

[b49] Zeljkovic I. *et al.* Mapping the unconventional orbital texture in topological crystalline insulators. Nat. Phys. 10, 572–577 (2014).

[b50] Ritschel T. *et al.* Orbital textures and charge density waves in transition metal dichalcogenides. Nat. Phys. 11, 328–331 (2015).

[b51] Xu X., Yao W., Xiao D. & Heinz T. F. Spin and pseudospins in layered transition metal dichalcogenides. Nat. Phys. 10, 343–350 (2014).

[b52] King P. D. C. *et al.* Quasiparticle dynamics and spinorbital texture of the SrTiO3 two-dimensional electron gas. Nat. Commun. 5, 3414 (2014). 24572991 10.1038/ncomms4414

[b53] Kokh K. A., Nenashev B. G., Kokh A. E. & Shvedenkov G. Y. Application of a rotating heat field in Bridgman-Stockbarger crystal growth. J. Cryst. Growth 275, E2129–E2134 (2005).

[b54] Perdew J. P., Burke K. & Ernzerhof M. Generalized gradient approximation made simple. Phys. Rev. Lett. 77, 3865–3868 (1996). 10062328 10.1103/PhysRevLett.77.3865

[b55] Kresse G. & Hafner J. *Ab initio* molecular dynamics for open-shell transition metals. Phys. Rev. B 48, 13115–13118 (1993). 10.1103/physrevb.48.13115 10007687

[b56] Kresse G. & Furthmüller J. Efficiency of ab-initio total energy calculations for metals and semiconductors using a plane-wave basis set. Comput. Mater. Sci. 6, 15–50 (1996). 10.1103/physrevb.54.11169 9984901

[b57] Blöchl P. E. Projector augmented-wave method. Phys. Rev. B 50, 17953–17979 (1994). 10.1103/physrevb.50.17953 9976227

[b58] Kresse G. & Joubert D. From ultrasoft pseudopotentials to the projector augmented-wave method. Phys. Rev. B 59, 1758–1775 (1999).

